# GFI1’s role in DNA repair suggests implications for tumour cell response to treatment

**DOI:** 10.15698/cst2018.07.149

**Published:** 2018-07-24

**Authors:** Charles Vadnais, Tarik Möröy

**Affiliations:** 1 Institut de recherches cliniques de Montréal (IRCM), Montréal, QC, Canada.; 2 Département de microbiologie, infectiologie et immunologie, Université de Montréal.; 3 Division of Experimental Medicine, McGill University, Montréal, QC, Canada.

**Keywords:** GFI1, DNA repair, DNA damage response, PRMT1, MRE11, 53BP1, Cytarabine, tumour therapy

## Abstract

Despite recent advances in cancer treatment through personalized and precision medicine and new avenues such as immunotherapy and chimeric antibodies, the induction of DNA damage either through irradiation or specific compounds remains the primary approach to kill tumour cells. Improvements in our understanding of how tumour cells respond to DNA damage, and especially how this response differs from that of normal cells, are crucial to the development of better and more efficient therapies. We have recently shown that the activity of the oncogenic transcription factor GFI1, which is required for the development and maintenance of T and B cell leukemia, increases the ability of tumour cells to repair their DNA following damage (Vadnais et al. Nat Commun 9(1):1418). GFI1 accomplishes this by regulating the post-translational modifications (PTM) of key DNA repair proteins, including MRE11 and 53BP1, by the methyltransferase PRMT1. Here, GFI1 acts as an accessory protein required for the interaction between the enzyme and its substrates. This has implications for the treatment response of tumour cells overexpressing GFI1, which includes T cell leukemia, neuroendocrine lung carcinomas and aggressive subtypes of medulloblastoma, and suggests that targeting GFI1's activity and with this its capacity to aid DNA repair may open avenues for new therapeutic approaches.

GFI1 was discovered over 20 years ago and a large body of experimental evidence defines its function as a DNA binding transcriptional repressor. Since GFI1 does not possess any enzymatic activity itself, it acts through the recruitment of enzymes that modify histone proteins to regulate the expression of its target genes. However, work with *Gfi1* KO mice showed that these animals had increased sensitivity to ionizing radiation; an effect that could not be explained solely by the absence of GFI1’s activity as a transcriptional regulator. Further analysis revealed that GFI1 deficient cells have a defect in the repair of DNA breaks and show delayed γ-H2AX signalling, while these processes were accelerated in cells overexpressing GFI1. A more detailed study by our laboratory finally clarified that GFI1 facilitates the repair of double strand DNA breaks (DSB) through homologous recombination (HR).

How is this achieved by a transcription factor? The analysis of RNA expression profiles of Gfi1 deficient and normal cells did not reveal any solid clues, but an experiment in which a Flag-tagged GFI1 protein was expressed in 293 T cells and GFI1 binding partners were identified by immunoprecipitation and mass spectrometry provided the answer: the list of GFI1 associated proteins from this and additional co-immunoprecipitation experiments showed that GFI1 forms complexes with the DNA repair proteins MRE11 and 53BP1, as well as the methyltransferase PRMT1. Subsequent biochemical analysis demonstrated that GFI1 is also required for the interaction between PRMT1 and both MRE11 and 53BP1 and, most importantly, for the addition of the "Asymmetric Dimethyl Arginine" (ADMA) mark on both proteins. In the case of MRE11, this methylation event is required for its exonuclease activity at sites of DSB, which is an initiating step in HR repair. Similarly to MRE11, GFI1 is required for PRMT1 to methylate 53BP1, which is, as MRE11, a key DDR protein that pro-motes the repair of DSB through Non-Homologous End Joining (NHEJ). Notably however, we found that GFI1 is not required for the enzymatic activity of PRMT1 per se, and that the overall methylation profile of GFI1 KO cells, or of GFI1 overexpressing cells, is not altered compared to their respective control cells. This implied that GFI1 is acting as a bridging factor or "enabler" for the interaction between PRMT1 and a subset of its targets (**Figure 1**). We hypothesize that this group of targets could contain more members than MRE11 and 53BP1 and is associated with specific biological functions that mediate GFI1's effect in the cellular response to DNA damage, as well as in other biological settings.

**Figure 1:**
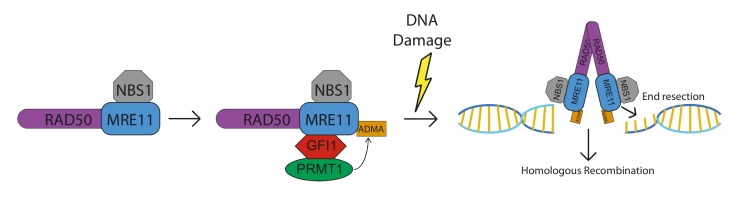
FIGURE 1: Model for the action of GFI1 in DNA repair. GFI1 acts as a bridge between the PRMT1 methyltransferase and key DNA repair proteins to promote their activity. Methylation of MRE11 and the corresponding facilitation of HR repair are shown as an example of this activity of GFI1.

According to our findings, this function of GFI1 is very likely independent of its capacity to bind DNA and thus constitutes a second, new mode of action of this protein. Indeed, treatment with benzonase which rids complexes of all remaining nucleic acids did not alter or affect the interaction between GFI1 and MRE11 and supports this view. Along this line, it is important to note that all available evidence suggests that GFI1's role in DNA repair occurs prior to the occurrence of damage and is independent of any interaction between GFI1 and sites of damage at the chromatin. Extensive immunofluorescence based experimentation with both endogenous and GFP-fusion GFI1 constructs showed no localization of GFI1 to the sites of DNA breaks. Furthermore, we found that the presence of the DNA binding domain of GFI1 was not required for its activity in promoting DNA repair. These results led us to propose the model where the role of GFI1 is a mediator or enabler to induce a specific steady state level of post-translational modifications on a set of key DNA repair proteins that favours the repair of damage once it occurs, and, by extension, the survival of the cells (**Figure 1**).

While our work has shown that GFI1 can function in HR, there is also strong evidence of its involvement in other repair pathways. For instance, the dynamics of γ-H2AX signalling are altered in GFI1 KO cells and this occurs in cells in all phases of the cell cycle, including G1, during which cells are unable to repair DSBs through HR in the absence of homologous template sequences. Similarly, comet assays carried out in cell populations where over >80% of cells were in the G1 phase showed large differences in the repair capacity in GFI1 KO cells, which is very unlikely to be due entirely to the fraction of cells in the S and G2 phases. In addition, while comet assays specific for DSB repair show the requirement of GFI1 for efficient repair, alkaline comet assays, which detect a much wider range of DNA damage events, including single stranded breaks and certain base alterations also show a strong effect of GFI1 expression on the repair capacity of cells, which is unlikely to be due only to HR repair of DSBs. Finally, the above mentioned IP-mass spectrometry experiment also indicated a complex formation between GFI1 and ATM and other DNA repair enzymes and kinases, but the mechanistic implications for these interactions remain to be determined. Given that the ATM kinase is involved in multiple repair pathways, this suggests an additional layer of regulation for the role of GFI1 in DNA repair.

Although not completely elucidated at a mechanistic level, the role of GFI1 in facilitating DNA repair clearly translates into a greater resistance of tumour cells to treatments that are based on induction of DNA damage such as radiation therapy or the use of chemotherapeutic drugs such as Cytarabine or Doxorubicin. Irradiation directly induces single and double strand breaks, while the pyrimidine nucleoside analogue Cytarabine is incorporated into DNA and causes strand breaks through a blockage of DNA replication. Similarly, Doxorubicin induces DNA damage through the inhibition of topoisomerase 2 and intercalation between bases, which leads to replication fork stalling. By comparison, Vincristine, also a commonly used anti-cancer therapeutic drug, does not act through the induction of DNA damage per se, but through the inhibition of mitosis by binding to tubulin and blocking chromosome separation during metaphase.

Our data show that leukemic cell lines overexpressing Gfi1 are more resistant to irradiation or Cytarabine and this increased resistance is also seen with treatment with Doxorubicin, but not with Vincristine (**Figure 2**). This is consistent with a role of GFI1 in rendering leukemic cells more resistant to treatment through increased capacity of repairing DNA damage and has direct implications for the treatment of GFI1 expressing tumours. Future studies, however, will have to give us a better understanding of the repair pathways on which GFI1 expressing tumours rely for their ability to respond to, and resist, radiotherapy and chemotherapy. In this regard, it is notable that GFI1 activity is not limited to hematopoietic tumours but also includes, among others, neuroendocrine lung carcinomas and certain aggressive subtypes of medulloblastoma. Many of these tumour types are common paediatric cancers, and while treatments do exist in several cases, their long-term side effects are considerable. A deeper understanding of treatment responses in these tumours will lead to both improvements in their efficacy and a reduction in their side effects and although difficult, targeting GFI1 for tumour therapy may prove to be a promising avenue in the future.

**Figure 2:**
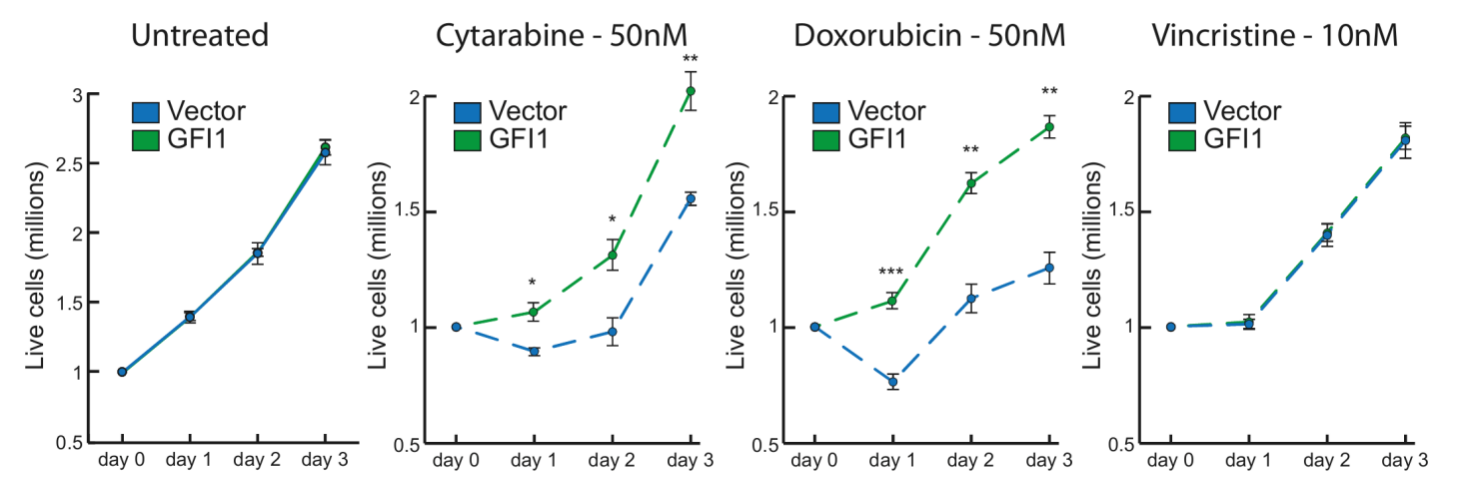
FIGURE 2: GFI1 overexpression protects from DNA damage based drug treatments. GFI1 overexpressing SupT1 cells and vector control cells were seeded at 1 million cells per ml and exposed to the indicated drugs. Cells were counted each following day. Average cell numbers are shown. * = p <0.05, ** = p<0,01, *** = p<0,001 on a Welch corrected T test.

